# Estimated visceral adiposity is associated with risk of cardiometabolic conditions in a population based study

**DOI:** 10.1038/s41598-021-88587-9

**Published:** 2021-04-27

**Authors:** Maria Ruiz-Castell, Hanen Samouda, Valery Bocquet, Guy Fagherazzi, Saverio Stranges, Laetitia Huiart

**Affiliations:** 1 Department of Population Health, Luxembourg Institute of Health, 1A-B, rue Thomas Edison, 1445 Strassen, Luxembourg; 2 Nutrition and Health Research Group, Department of Population Health, Luxembourg Institute of Health, 1A-B, rue Thomas Edison, 1445 Strassen, Luxembourg; 3 Competence Centre for Methodology and Statistics, Department of Population Health, Luxembourg Institute of Health, 1A-B, rue Thomas Edison, 1445 Strassen, Luxembourg; 4 Deep Digital Phenotyping Research Unit, Department of Population Health, Luxembourg Institute of Health, 1A-B, rue Thomas Edison, 1445 Strassen, Luxembourg; 5 Department of Epidemiology and Biostatistics, Schulich School of Medicine and Dentistry, Western University, Kresge Building, 1151 Richmond St, London, ON N6A 3K7 Canada; 6 Department of Family Medicine, Schulich School of Medicine and Dentistry, Western University, Western Centre for Public Health and Family Medicine, 1465 Richmond St, London, ON N6G 2M1 Canada

**Keywords:** Metabolic disorders, Epidemiology

## Abstract

Visceral adiposity is a major risk factor of cardiometabolic diseases. Visceral adipose tissue (VAT) is usually measured with expensive imaging techniques which present financial and practical challenges to population-based studies. We assessed whether cardiometabolic conditions were associated with VAT by using a new and easily measurable anthropometric index previously published and validated. Data (1529 participants) came from the European Health Examination Survey in Luxembourg (2013–2015). Logistic regressions were used to study associations between VAT and cardiometabolic conditions. We observed an increased risk of all conditions associated with VAT. The total adjusted odds ratio (AOR, [95% CI]) for hypertension, prediabetes/diabetes, hypercholesterolemia, and hypertriglyceridemia for the fourth quartile of VAT compared to the lowest were 10.22 [6.75, 15.47]), (5.90 [4.02, 8.67]), (3.60 [2.47, 5.25]) and (7.67 [5.04, 11.67]. We observed higher odds in women than in men for all outcomes with the exception of hypertension. Future studies should investigate the impact of VAT changes on cardiometabolic health and the use of anthropometrically predicted VAT as an accurate outcome when no biomedical imaging is available.

## Introduction

Over the past 20 years there has been a significant global increase in the prevalence of cardiovascular (CVD) and metabolic conditions such as hypertension, type 2 diabetes, hypercholesterolemia, hypertriglyceridemia and metabolic syndrome^1–4^. Despite downward trends in mortality rates due to CVD in high-income countries over the last decades, new evidence suggests a possible plateau in CVD mortality in recent years^5^. This has contributed to cardiovascular diseases being the leading cause of mortality and early morbidity worldwide^5,6^ with nearly 30% of global causes of death due to CVD diseases^7^.

An excess of visceral fat is linked to chronic diseases such as metabolic and cardiovascular conditions emphasizing the strong association between cardiometabolic health and excess of ectopic fat^8^. Adipose tissue is a complex organ with different roles on energy metabolism, endocrine function and inflammation^9,10^. A malfunction produced by an excess of adipose tissue is associated with metabolic disorders^11^. In particular, an excess of ectopic fat, located in the abdominal cavity surrounding the organs, also known as visceral adipose tissue (VAT), is considered a major risk factor for metabolic and cardiovascular diseases, independently of general adiposity^9,12^. VAT can be accurately measured through biomedical imaging techniques such as magnetic resonance imaging (MRI) and computed tomography-scan (CT-Scan)^13^. However, these techniques are expensive, limited to specialised medical use and show high exposures to radiation (e.g. CT-Scan)^13^, thus being ineffective for population-based studies. Due to those limitations, simple anthropometric measurement (e.g. waist circumference (WC), waist-to-hip circumference ratio) are used for the indirect assessment of visceral adiposity in both clinical and population-based studies^14–16^. Nevertheless, these measurements have the major limitation of not being able to differentiate between VAT and subcutaneous abdominal adipose tissue (SAAT). Recently, Samouda et al. suggested a more accurate anthropometric prediction of VAT by subtracting the most correlated anthropometric measurement with SAAT from an abdominal anthropometric measurement highly correlated with total abdominal adipose tissue and the most possible correlated with VAT^17^. Using this equation, VAT can be readily predicted by combining a small number of easy to measure anthropometric features (e.g. WC, proximal thigh circumference and body mass index (BMI)). The anthropometric predictive models of VAT developed by Samouda et al. in 2013, were validated by Brown et al. in 2017 and 2018. They observed that compared to BMI and WC, predicted models of VAT were the most accurate predictors of cardiometabolic conditions as well as all-cause and cause-specific mortality (e.g. CVD, cancer) in Europid descendants from the Third National Health and Nutrition Examination Survey, 1988–1994 (NHANES III), when biomedical imaging data are not available or feasible^18,19^.

Results from the European Health Examination Survey in Luxembourg (EHES-LUX) have shown that the prevalence of hypertension and obesity is high in the country, with one third of the population showing hypertension and obesity rates reaching 20%^20,21^. Despite having information on the health situation, up-to-date and exhaustive characterizations of the cardiometabolic health are still missing for the Luxembourgish population, particularly in regards to specific risk factors such as VAT accumulation.

The present study aims to assess whether anthropometrically predicted visceral adipose tissue was associated with hypertension, prediabetes and diabetes, as well as hypercholesterolemia and hypertriglyceridemia, after adjusting for socio-demographic and behavioural characteristics in a population-based study.

## Results

### Participants’ characteristics

Nearly half of participants were women (52%) (Supplementary Table S1). Differences were observed between men and women. Men were more likely to have higher BMI, WC, and anthropometrically predicted VAT while women had higher thigh circumference. Men were more likely to have higher systolic and diastolic blood pressure, LDL cholesterol, triglycerides and fasting plasma glucose levels. Men had almost twice as much hypertension (40.09%), combined prediabetes and diabetes (42.63%) and hypertriglyceridemia (40.17%) as in women (23.40%, 22.16% and 19.09% respectively). Women smoked and consumed alcoholic drinks less than men.

### Cardiometabolic conditions by quartiles of VAT

The proportion of hypertension, combined prediabetes and diabetes, hypertriglyceridemia and metabolic syndrome increased with VAT quartiles in both men and women (Table 1). The largest prevalence gradient was observed for metabolic syndrome in both men (from 1.7% in the first quartile to 82.1% observed in the fourth quartile) and women (from 1.1% in the first quartile to 62.2% observed in the fourth quartile). The proportion of hypercholesterolemia increased with VAT in both men and women. We observed that median values of WC and thigh circumference increased with VAT, but when we visualized VAT for similar WC values (Supplementary Figure S1), we observed that thigh circumference decreased for both men and women. Similar results were observed in the prevalence of cardiometabolic conditions by quartiles of WC (Supplementary Table S2).

**Table 1:** Participants’ characteristics by anthropometrically-predicted visceral adipose tissue (VAT) quartiles stratified by sex: European Health Examination Survey in Luxembourg, 2013–2015 (N = 1441).

	Q1	Q2	Q3	Q4	*P* for trend^c^
Men, n	173	173	173	173	
**Cardiometabolic conditions, n (%)**					
Hypertension	26 (15.0)	55 (32.0)	76 (43.9)	120 (69.4)	< 0.001
Prediabetes and diabetes	40 (23.1)	61 (35.5)	77 (44.5)	113 (65.3)	< 0.001
Hypercholesterolemia^a^	100 (57.8)	136 (79.1)	134 (77.5)	136 (78.6)	< 0.001
High LDL-cholesterol^b^	107 (61.9)	136 (78.6)	122 (70.5)	107 (61.9)	NS
Low HDL-cholesterol^c^	46 (26.6)	51 (29.5)	56 (32.4)	73 (42.2)	< 0.001
Hypertriglycemia	31 (17.9)	57 (33.0)	78 (45.1)	112 (64.7)	< 0.001
Metabolic Syndrome	3 (1.7)	36 (20.8)	89 (51.5)	142 (82.1)	< 0.001
**Cardiometabolic risk factors, median (P25, P75)**					
Systolic blood pressure, mm Hg	123.0 (117.0, 131.0)	125.5 (120.2, 134.2)	131.5 (124.5, 140.0)	135.0 (126.5, 145.0)	< 0.001
Diastolic blood pressure, mm Hg	76.0 (71.0, 82.5)	82.0 (75.0, 87.0)	84.5 (79.7, 89.7)	87.5 (80.5, 94.0)	< 0.001
Total cholesterol, mg/dL	194.0 (177.0, 219.0)	204.0 (184.0 (229.0)	209.0 (186.0, 233.0)	197.0 (170.0, 233.0)	NS
HDL-cholesterol, mg/dL	48.0 (39.0, 58.0)	45.0 (39.0, 55.0)	43.0 (38.0, 50.0)	40.0 (36.0, 48.0)	< 0.001
LDL-cholesterol, mg/dL	123.0 (106.0, 151.0)	133.0 (121.0, 158.0)	136.0 (111.0, 160.0)	125.0 (101.0, 152.0)	NS
Triglycerides, mg/dL	87.0 (67.0, 123.0)	102.0 (75.0, 146.0)	127.0 (90.0, 175.0)	143.0 (105.0, 216.0)	< 0.001
Fasting plasma glucose, mg/dL	94.0 (88.0, 99.0)	96.0 (91.0, 103.0)	98.0 (93.0, 104.0)	105.0 (97.0, 114.0)	< 0.001
Haemoglobin A1c, mol/mol	36.0 (33.0, 38.0)	37.0 (34.0, 39.0)	37.0 (34,0, 40,0)	39.0 (37.0, 43.0)	< 0.001
**Anthropometric characteristics (median (P25, P75))**					
BMI, kg/m^2^	23.8 (22.3, 25.5)	26.1 (23.9, 27.6)	27.8 (25.9, 29.6)	30.9 (28.7, 34.4)	< 0.001
Waist circumference, cm	84.6 (80.6, 88.2)	92.1 (89.0, 95.4)	98.5 (95.0, 103.0)	111.0 (105.0, 117.0)	< 0.001
Thigh circumference, cm	57.5 (55.1, 61.0)	58.5 (55.7, 61.5)	60.0 (56.5, 64.0)	61.5 (58.5, 66.0)	< 0.001
**Demographic characteristics, median (P25, P75)**					
Age, years	36.2 (31.4, 42.7)	43.4 (37.8, 49.6)	47.7 (41.1, 54.4)	53.1 (47.9, 58.4)	< 0.001
**Lifestyles and socioeconomic characteristics, n (%)**					
Current smoking or quit < 12 months (vs non-smokers or quit > 12 months)	46 (26.7)	49 (28.3)	44 (25.4)	45 (26.0)	NS
Current drinkers (vs no drinkers)	169 (97.7)	169 (97.7)	168 (97.1)	170 (98.3)	NS
Alcohol consumption					
Non-alcohol (weekly) consumption	52 (30.0)	45 (26.0)	44 (25.4)	34 (19.6)	
≤ 6 drinks/week	61 (35.3)	58 (33.5)	52 (30.1)	50 (28.9)	
> 6 drinks/week	60 (34.7)	70 (40.5)	77 (44.5)	89 (51.5)	
Aerobic PA > = 150 min (vs APA < 150 min)	92 (53.2)	77 (44.5)	61 (35.3)	41 (23.8)	< 0.001
Aerobic PA, min /week (median (P25, P75))	180.0 (30.0, 360.0)	120.0 (0.0, 260.0)	80.0 (0.0, 200.0)	0.0 (0.0, 120.0)	< 0.001
Tertiary education (vs 2ary and Primary)	86 (49.7)	81 (47.1)	49 (28.3)	48 (27.9)	< 0.001
Working (vs not working)	157 (90.8)	151 (87.8)	136 (78.6)	121 (69.9)	< 0.001
Women, n	188	187	186	188	
**Cardiometabolic conditions, n (%)**					
Hypertension	13 (6.9)	27 (14.4)	45 (24.3)	90 (47.8)	< 0.001
Prediabetes and diabetes	14 (7.5)	22 (11.8)	45 (24.3)	82 (43.6)	< 0.001
Hypercholesterolemia^a^	88 (46.8)	107 (57.5)	142 (76.8)	159 (84.6)	< 0.001
High LDL-cholesterol^b^	77 (41.0)	103 (55.1)	127 (68.3)	130 (69.2)	< 0.001
Low HDL-cholesterol^c^	37 (19.7)	40 (21.4)	49 (26.3)	81 (43.1)	< 0.001
Hypertriglycemia	7 (3.7)	12 (6.4)	43 (23.1)	81 (43.1)	< 0.001
Metabolic syndrome	2 (1.1)	13 (7.0)	57 (31.0)	117 (62.6)	< 0.001
**Cardiometabolic risk factors, median (P25, P75)**					
Systolic blood pressure, mm Hg	108.5 (103.0, 116.0)	113.5 (106.5, 123.0)	120.8 (111.0, 131.0)	124.7 (113.5, 136.0)	< 0.001
Diastolic blood pressure, mm Hg	72.5 (67.5, 77.5)	75.0 (69.0, 81.5)	78.3 (74.0, 84.5)	82.5 (75.5, 87.5)	< 0.001
Total cholesterol, mg/dL	184.5 (160.0, 208.5)	195.0 (175.0, 226.0)	205.0 (184.0, 227.0)	208.5 (184.0, 228.5)	< 0.001
HDL-cholesterol, mg/dL	59.0 (51.0, 67.0)	60.0 (51.0, 70.0)	58.0 (49.0, 66.0)	52.0 (42.0, 60.5)	< 0.001
LDL-cholesterol, mg/dL	109.5 (93.0, 128.0)	121.0 (102.0, 141.0)	129.0 (109.0, 147.0)	132.0 (108.0, 152.0)	< 0.001
Triglycerides, mg/dL	68.0 (53.0, 82.5)	69.0 (55.0, 91.0)	82.0 (64.0, 110.0)	103.5 (80.5, 139.5)	< 0.001
Fasting plasma glucose, mg/dL	89.0 (84.0, 93.0)	92.0 (86.0, 95.0)	93.0 (88.0, 99.0)	98.0 (91.5, 105.0)	< 0.001
Haemoglobin A1c, mol/mol	34.0 (33.0, 37.0)	36.0 (33.0, 38.0)	37.5 (34.0, 39.0)	39.0 (37.0, 42.0)	< 0.001
**Anthropometric characteristics (median (P25, P75))**					
BMI, kg/m^2^	21.3 (19.8, 22.7)	23.4 (21.7, 25.3)	25.8 (23.6, 27.8)	31.6 (28.2, 35.2)	< 0.001
Waist circumference, cm	73.0 (70.0, 76.8)	80.0 (75.4 ,84.0)	87.9 (83.0, 92.0)	101.0 (94.4, 108.5)	< 0.001
Thigh circumference, cm	57.5 (54.0, 61.5)	59.8 (56.0, 63.5)	61.0 (56.5, 64.5)	64.2 (59.1, 69.3)	< 0.001
**Demographic characteristics, median (P25, P75)**					
Age, years	35.2 (30.2, 39.9)	43.0 (36.8, 49.1)	50.6 (43.1, 56.3)	52.9 (45.8, 59.1)	< 0.001
**Lifestyles and socioeconomic characteristics, n (%)**					
Current smoking or quit < 12 months (vs non-smokers or quit > 12 months)	40 (21.3)	42 (22.5)	36 (19.5)	42 (22.5)	NS
Alcohol consumption					
Current drinkers (vs no drinkers)	180 (95.7)	174 (93.1)	166 (89.7)	162 (86.6)	< 0.001
Non-alcohol (weekly) consumption	102 (54.3)	99 (52.9)	89 (48.1)	110 (59.1)	
≤ 6 drinks/week	70 (37.2)	56 (30.0)	60 (32.4)	48 (25.8)	
> 6 drinks/week	16 (8.5)	32 (17.1)	36 (19.5)	28 (15.1)	
Aerobic PA > = 150 min (vs APA < 150 min)	87 (46.3)	71 (38.0)	65 (35.1)	44 (23.5)	< 0.001
Aerobic PA, min/week (median (P25, P75))	120.0 (0.0, 240.0)	90.0 (0.0, 210.0)	60.0 (0.0, 210.0)	0.0 (0.0, 120.0)	< 0.001
Tertiary education (vs 2ary and Primary)	114 (60.6)	74 (39.8)	48 (26.0)	27 (14.4)	< 0.001
Working (vs not working)	164 (87.2)	141 (75.4)	129 (69.4)	102 (54.3)	< 0.001

Missing values range from 1 to 14 observations; Q: quartile.Q1 (≤ 113.84), Q2 (113.85—151.59), Q3 (151.60- 196.65), and Q4 (≥ 196.66) for men and Q1 (≤ 58.78), Q2 (58.79- 89.38), Q3 (89.39- 127.35), and Q4 (≥ 127.36) for women.

*n* number, *∆Low HDL* cholesterol was defined as blood as HDL-C less than 50 mg/dL for women and 40 mg/dL for men.

^a^Hypercholesterolemia was defined as total cholesterol ≥ 190 mg/dL or on medication to reduce cholesterol.

^b^High LDL-Cholesterol was defined as blood LDL-C ≥ 115 mg/dL.

^c^Cochran-Armitage trend test fo categorical variables and Jonckheere-Terpstra test for continuous variables.

### VAT association with all cardiometabolic conditions

Results from logistic regression analysis examining the association between anthropometrically predicted VAT and cardiometabolic conditions are presented in Table 2. We observed an increase in the odds of all metabolic and cardiovascular conditions associated with VAT in both men and women. The strength of the association was reduced but remained statistically significant after adjusting for socioeconomic status (education and employment status) (model 1), and lifestyle (model 2). The association observed was strongest in men for hypertension: adjusted OR [95% CI] for men were 2.51 [1.46, 4.29], 4.08 [2.40, 6.93], and 11.83 [6.82, 20.49] for the second, third and fourth quartile of VAT. For women, the values were 1.93 [0.92, 4.00], 3.41 [1.69, 6.85], and 8.21 [4.12, 16.36] for the second, third and fourth quartile of VAT. Nevertheless, women observed a strongest association for combined prediabetes and diabetes 7.57 [3.93, 14.59] for the fourth quartile of VAT in women compared to 5.41 [3.26, 8.97 in men), hypercholesterolemia (5.28 [3.09, 9.00] for the fourth quartile of VAT in women compared to 2.26 [1.33, 3.84] in men) and hypertriglyceridemia (14.62 [6.30, 33.90] for the fourth quartile of VAT in women compared to 6.78 [3.97, 11.56] in men.

**Table 2:** Results of logistic regression measuring the association between cardiometabolic conditions (hypertension, prediabetes/diabetes, hypercholesterolemia, hypertriglycemia) and anthropometrically predicted visceral adiposity in models unadjusted and adjusted for participants’ lifestiles and socioeconomic characteristics: European Health Examination Survey in Luxembourg, 2013–2015 (N = 1441).

VAT quartiles	Total				Men			Women		
	Model 1^a^ OR [95% CI]	Model 2^b^ AOR [95% CI]	Model 3^c^ AOR [95% CI]		Model 1^a^ OR [95% CI]	Model 2^b^ AOR [95% CI]	Model 3^c^ AOR [95% CI]	Model 1^a^ OR [95% CI]	Model 2^b^ AOR [95% CI]	Model 3^c^ AOR [95% CI]
**Hypertension**										
Q1	1.00	1.00	1.00		1.00	1.00	1.00	1.00	1.00	1.00
Q2	2.41 [1.58, 3.69]	2.39 [1.55, 3.68]	2.30 [1.50, 3.51]		2.53 [1.47, 4.34]	2.48 [1.45, 4.27]	2.51 [1.46, 4.29]	2.41 [1.18, 4.90]	2.01 [0.97, 4.16]	1.93 [0.92, 4.00]
Q3	4.28 [2.84, 6.44]	4.11 [2.69, 6.26]	3.89 [2.56, 5.90]		4.32 [2.55, 7.32]	4.09 [2.40, 6.95]	4.08 [2.40, 6.93]	4.65 [2.38, 9.05]	3.49 [1.75, 6.98]	3.41 [1.69, 6.85]
Q4	11.83 [7.90, 17.72]	11.42 [7.48, 17.42]	10.22 [6.75, 15.47]		13.22 [7.68, 22.76]	11.96 [6.91, 20.69]	11.83 [6.82, 20.49]	13.16 [6.91, 25.04]	8.78 [4.42, 17.42]	8.21 [4.12, 16.36]
**Prediabetes/diabetes**										
Q1	1.00	1.00	1.00		1.00	1.00	1.00	1.00	1.00	1.00
Q2	1.66 [1.13, 2.45]	1.68 [1.13, 2.50]	1.56 [1.05, 2.31]		1.75 [1.08, 2.84]	1.75 [1.07, 2.85]	1.68 [1.03, 2.74]	1.69 [0.83, 3.46]	1.55 [0.75, 3.18]	1.47 [0.71, 3.03]
Q3	2.88 [1.99, 4.18]	2.81 [1.91, 4.13]	2.60 [1.77, 3.81]		2.66 [1.65, 4.29]	2.58 [1.60, 4.18]	2.43 [1.49, 3.95]	3.82 [2.00, 7.30]	3.32 [1.71, 6.41]	3.16 [1.62, 6.15]
Q4	6.50 [4.51, 9.35]	6.72 [4.56, 9.90]	5.90 [4.02, 8.67]		6.23 [3.83, 10.10]	6.09 [3.71, 10.00]	5.41 [3.26, 8.97]	9.35 [5.00, 17.47]	7.66 [3.98, 14.77]	7.57 [3.93, 14.59]
**Hypercholesterolemia**										
Q1	1.00	1.00	1.00		1.00	1.00	1.00	1.00	1.00	1.00
Q2	2.02 [1.47, 2.75]	1.94 [1.41, 2.65]	1.90 [1.39, 2.61]		2.93 [1.79, 4.78]	2.89 [1.76, 4.74]	2.78 [1.69, 4.58]	1.51 [1.00, 2.29]	1.40 [0.91, 2.14]	1.38 [0.90, 2.12]
Q3	3.16 [2.27, 4.39]	2.91 [2.07, 4.08]	2.81 [2.00, 3.97]		2.67 [1.65, 4.31]	2.49 [1.52, 4.08]	2.30 [1.39, 3.79]	3.79 [2.41, 5.95]	3.37 [2.11, 5.37]	3.27 [2.03, 5.26]
Q4	4.27 [3.02, 6.03]	3.71 [2.57, 5.34]	3.60 [2.47, 5.25]		3.02 [1.85, 4.91]	2.66 [1.60, 4.43]	2.26 [1.33, 3.84]	6.28 [3.83, 10.31]	5.17 [3.05, 8.78]	5.28 [3.09, 9.00]
**Hypertriglycemia**										
Q1	1.00	1.00	1.00		1.00	1.00	1.00	1.00	1.00	1.00
Q2	2.01[1.29, 3.12]	2.01 [1.28, 3.13]	1.81 [1.15, 2.82]		2.21 [1.32, 3.70]	2.20 [1.31, 3.71]	2.08 [1.22, 3.55]	1.97 [0.75, 5.19]	1.72 [0.64, 4.60]	1.62 [0.60, 4.36]
Q3	4.31 [2.86, 6.50]	4.09 [2.68, 6.26]	3.66 [2.41, 5.57]		3.68 [2.22, 6.08]	3.41 [2.05, 5.66]	3.18 [1.90, 5.30]	8.50 [3.67, 19.67]	7.00 [3.01, 16.28]	6.61 [2.80, 15.60]
Q4	9.56 [6.39, 14.32]	9.43 [6.17, 14.42]	7.67 [5.04, 11.67]		8.36 [5.02, 13.93]	7.72 [4.59, 12.98]	6.78 [3.97, 11.56]	20.83 [9.18, 47.25]	15.44 [6.69, 35.61]	14.62 [6.30, 33.90]

Total also adjusted for sex.

*AOR* adjusted odds ratio, *CI* confidence interval, *OR* odds ratio, *VAT* anthropometrically-predicted visceral adipose tissue, *Q* quartile.

^a^Unadjusted model.

^b^Model 1 adjusted for education and employment status.

^c^Model 2 adjusted for smoking status, alcohol consumption and aerobic physical activity.

## Discussion

The present study highlighted an increase of all metabolic and cardiovascular conditions associated with anthropometrically predicted VAT in adults aged 25–64. The association observed was independent of socioeconomic status and lifestyles. Our findings confirm that VAT is a major independent predictor risk factor of cardiometabolic risk as observed in previous epidemiological studies^22^. This can be explained by the high metabolic activity of VAT and its pro-inflammatory activity (production of cytokines with inflammatory effects and blocking of those anti-inflammatory)^23,24^. Moreover, compared to other fat deposits, VAT has larger and dysfunctional adipocytes, which are less insulin sensitive and with increased lipolytic activity. As the adipocytes grow, they accumulate triglycerides, becoming leptin resistant and promoting the synthesis and release of free fatty acids^23^. We observed that both WC and thigh circumference increased with VAT, but as previously reported, when WC and age were constant thigh circumference decreased with VAT for both men and women^25^. This is in line with previous evidence showing that VAT is the major risk factor of cardiometabolic morbidity and premature mortality, while lower-body fat mas plays a protective role and should be maintained when reducing VAT^26^. We observed sex differences in cardiometabolic conditions with men having a higher prevalence of all conditions compared to women, in line with previous evidence among middle-aged adults^27,28^. Hypertension, combined diabetes and prediabetes and hypertriglyceridemia prevalence were almost twice as high in men compared to women. Metabolic syndrome was 1.5 times higher in men compared to women. Closely related to these results are differences observed in WC and VAT being both higher in men compared to women as well as certain risk behaviours and socioeconomic differences such as lower consumption of alcohol and cigarettes and lower socioeconomic status in women compared to men. Sex differences on VAT are expected, since men are characterized by having a greater concentration of fat in the abdominal area compared to women that usually concentrates in the thighs and hip (gluteo-femoral pattern)^29^. Luxembourg is a small European country whose cultural diversity—nearly one in two residents is of foreign origin—accounts for the country’s well-documented heterogeneous and complex health profile. As in other high-income countries, in Luxembourg CVD is the leading cause of death (31.8%) while deaths due to endocrine, nutritional and metabolic diseases have more than doubled in the past 15 years, in both men and women^30,31^. Results from the present study show that compared to a previous study conducted in 2007 in Luxembourg, no reduction in cardiometabolic conditions has been observed over the last decade^32^ and even an increase has been noted in certain conditions such as diabetes or metabolic syndrome^33^. This could explain why cardiovascular diseases remained the main cause of mortality in Luxembourg in 2016^30^. These results provide compelling evidence on the current burden of cardiovascular and metabolic conditions in Luxembourg in both men and women, and the need for public health initiatives to alleviate the societal impact of these highly prevalent disease conditions. Moreover, VAT management should be considered as a privilege area of study to tackle metabolic and cardiovascular health issues. As reported in other studies^34,35^, we observed that cardiometabolic conditions were more prevalent among individuals with poor nutritional status, smoking, consuming alcohol, and with sedentary habits. As observed by Shi et al. 2011, abstention from smoking, regular physical activity, and a moderate consumption of alcohol were related with less cardiometabolic conditions^34^. Moreover, studies have observed that a combination of a healthy diet and physical activity (regardless of the quantity/intensity) have a strong effect on reducing VAT, even with minimal weight loss, thus representing a cost effective non-pharmacological intervention to reduce the impact of VAT on cardiometabolic health^22^. At present, there is no specific treatment to reduce VAT without also reducing lower-body fat mass. There are only experimental clinical studies in progress with the aim of personalizing treatments to each specific situation/individual^36,37^. Studies also observed an effect of socioeconomic conditions, with those with lower socioeconomic status being at higher risk of developing cardiometabolic diseases^38–40^. Both lifestyle and socioeconomic characteristics explained in part, but not completely, the association between VAT and cardiometabolic conditions, as we observed that the association remained statistically significant even after adjusting for those factors. Although there is evidence showing that the socioeconomic effect could be mediated by health behaviours (e.g. smoking)^35^, we observed two independent effects (model 1 and model 2).

Results of this study must be interpreted with caution, taking into account the following limitations. The design of the present study was cross-sectional, hence no temporal relationship or causality can be inferred. The participation rate was rather low yet still representative of the target population^41^. VAT was measured indirectly. Instead of using biomedical imaging techniques (e.g. MRI, CT-Scan), we estimated VAT with anthropometric measurements. Nevertheless, the predictive anthropometric models of VAT used in the present study were previously developed and validated as the most accurate predictor of biological cardiometabolic risk factors, all-cause and cause-specific mortality in Europid descendants, when biomedical imaging data are not available^17–19^. Results from these studies observed a high correlation of VAT (assessed by imaging techniques) with anthropometric VAT models, whereas other studies observed that WC was higher correlated with SAT and fat mass than with VAT^42^. Finally, we did not have information on other potential biomarkers of cardiometabolic risk such as markers of inflammation. In summary, anthropometrically predicted VAT was associated in the present work with all metabolic and cardiovascular conditions in both men and women even after adjusting for socio-demographic and behavioural characteristics. This reinforces the role played by VAT as a major independent risk factor for cardiometabolic health. Anthropometrically predicted VAT should be used in future epidemiological studies to investigate metabolic and cardiovascular disease when no biomedical imaging measurements are available and replicated in other contexts /populations. Likewise, prospective and intervention studies should place greater focus on the impact of changes in VAT on cardiometabolic health.

## Methods

### Study population

Data for the present study came from EHES-LUX, a cross-sectional population based survey done in 2013–2015. The study was performed following a one-stage sampling procedure stratified by age, sex, and district of residence. Residents in Luxembourg aged 25–64 years old were invited randomly to participate in the survey with the exception of those individuals living in institutions such as hospitals, nursing homes or prisons. A total of 1529 individuals participated in the study and signed an informed consent^20,41^. The survey consisted in 3 sections: a health questionnaire, a medical examination and the collection of biological samples. The questionnaire and examination were performed by trained nurses in German, French, Portuguese and/or English. The analysis of biological samples was performed in a National certified laboratory. Out of all participants, 21 were pregnant women (excluded from the present analysis) and 1469 underwent biological analysis. A total of 1448 individuals had complete information in the three health sections of the survey (94.7%). While objective measures of VAT (e.g. CT-Scan, MRI) are not covered by EHES-LUX, the survey does dispose of accurate and complete set of anthropometric measurements. In the present study we excluded 5 individuals with values of visceral adipose tissue inferior or equal to zero. One individual did not have a measure of height and thus VAT was not possible to calculate. The final sample size of the present study was 1441 participants. The study was approved by the National Research Ethics Committee (CNER, No. 201205/07) and notified to the Luxemburgish National Commission for Data Protection (CNPD). All methods were performed in accordance with the relevant guidelines and regulations.

### Cardiovascular and metabolic conditions

Hypertension was defined as systolic/diastolic blood pressure of ≥ 140/90 mm Hg, self-report of a physician diagnosis, or on antihypertensive medication^41^. Fasting plasma glucose, haemoglobin A1c, total cholesterol, high-density lipoprotein cholesterol (HDL-C), low-density lipoprotein cholesterol (LDL-C) and triglycerides were measured in participant’s blood samples^41^. Prediabetes and diabetes were defined as fasting glucose of ≥ 100 mg/dL, self-report of physician diagnosis, or on antidiabetic medication. Hypercholesterolemia was defined as total cholesterol ≥ 190 mg/dL or on medication to reduce cholesterol. High LDL-C was defined as LDL-C ≥ 115 mg/dL for both men and women^43^. Low HDL-C was defined as HDL-C < 50 mg/dL for women and 40 mg/dL for men. In this paper, we use the term hypercholesterolemia in reference to total cholesterol. Hypertriglycemia was defined as triglycerides ≥ 150 mg/dL or on medication. Metabolic Syndrome was defined following the International Diabetes Federation^44^. This definition includes the presence of central obesity WC ≥ 94 cm for men and WC ≥ 80 cm for women) and at least two of the following factors: total triglycerides ≥ 150 mg/dL or on medication, low HDL-C (< 40 mg/dL for men, < 50 mg/dL for women) or on medication, high blood pressure (systolic blood pressure ≥ 130 mm Hg or diastolic blood pressure ≥ 85 mm Hg) or on medication, high fasting plasma glucose (≥ 100 mg/dL) or self-report of a physician diagnosis.

### Anthropometric variables

Weight and height, together with waist, hip and thigh circumferences were measured by trained nurses following Lohman recommendations^45^. BMI was calculated as weight divided by height squared (kg/m^2^). Estimated VAT was calculate as: 6*waist circumference − 4.41*proximal thigh circumference + 1.19*age – 213.65 for men and as 2.15*waist circumference – 3.63*proximal thigh circumference + 1.46*age + 6.22* BMI − 92.713 for women^17^. The equations used to estimate VAT are based on the strong correlation observed between thigh circumference and subcutaneous fat, as assessed by CT-Scan. The anthropometric VAT model assumed that by subtracting the most correlated anthropometric measurement with SATT from the most correlated anthropometric measurement with total abdominal and VAT as assessed by CT-Scan (WC), we can obtain the most accurate prediction of VAT by anthropometry. The population used to develop the equation was 253 individuals aged 18–78 years old, from Southern Europe, and with BMI values ranged between 16 and 53 kg/m^2^. Multiple linear regressions with an empirical selection of the variables were performed and validated. Model variances, collinearity, and errors (e.g. Bland and Altman plots representation) were assessed. Sensitivity and specificity of the anthropometric models for the diagnosis of visceral adiposity excess in a clinical setting, along with the positive and negative predictive value of the models for predicting a cut-off of 130 cm2, were also assessed. Models were validated in a second sample of 139 participants (77 women, BMI range: 19.25–47.96 kg/m^2^ and 62 men, BMI range: 18.55–52.94 kg/m^2^)^17^. Models were further validated as predictors of cardiometabolic conditions, cancer and early death in 10.624 participants from the US National Health and Nutrition Examination Survey (NHANES), followed for 20 years^18,19^.

### Covariates

Based on the literature review we selected a list of potential covariates^27,34,38^. Demographic characteristics included age and sex (men and women). Lifestyle characteristics included smoking status (current smoking or quit < 12 months vs non-smokers or quit > 12 months), alcohol consumption (non-alcohol consumption, ≤ 6 drinks/week, > 6 drinks/week) and aerobic physical activity (min/week). For both alcohol and physical activity, we used validated questionnaires with standardized questions for European populations from the European Health Interview Survey (EHIS). Socioeconomic characteristics included education (tertiary education vs secondary and primary), and job status (employed vs not employed).

### Statistical data analysis

We calculated VAT score quartiles (Q) based on statistical distribution (data-driven), clinical utility and for comparison with most previous studies using the same approach, for both men and women as follows: Q1 (≤ 113.84), Q2 (113.85–151.59), Q3 (151.60–196.65), and Q4 (≥ 196.66) for men and Q1 (≤ 58.78), Q2 (58.79–89.38), Q3 (89.39–127.35), and Q4 (≥ 127.36) for women. We test normality with the Kolmogorov–Smirnov test. Medians were used for continuous variables and frequencies for categorical variables. To analyse associations between cardiometabolic outcomes (e.g. hypertension, prediabetes and diabetes and total cholesterol) and covariates, we used a Pearson's chi-squared test (for probabilities related to frequencies) or Wilcoxon–Mann–Whitney U two-sample test (for probabilities related to medians) to compare characteristics between men and women. We used non parametric test because data was not normally distributed. Distributions of cardiometabolic conditions across VAT quartiles were measured with the Cochran-Armitage P-trend test for categorical variables and Jonckheere-Terpstra test for continuous variables. We performed multivariable logistic regression analyses to study the association between VAT quartiles and cardiometabolic outcomes in unadjusted (Model 1) and adjusted models for education and employment status (Model 2), and lifestyle (e.g. smoking, alcohol consumption and physical activity) and socioeconomic conditions (Model 3). All analyses were stratified by sex, given the well-known differences in visceral adiposity distribution and cardiometabolic disease prevalence between women and men^46^. Only variables with a P-value < 0.20 in univariate analyses were included in the final model. Although the main objective of the paper was to analyze VAT quartiles related to cardiometabolic outcomes, we performed additional analyses dividing individuals by quartiles of waist circumference. The aim was to assess whether the results were similar, better, or worse than those obtained with estimated VAT (Supplementary Table S2).We used the Akaike information criterion (AIC) to evaluate the model fit quality of the univariate analyses using VAT and WC quartiles. We calculated WC score quartiles for both men and women as follows: Q1 (≤ 88.50), Q2 (88.51–95.45), Q3 (95.46–103.99), and Q4 (≥ 104.00) for men and Q1 (≤ 76.00), Q2 (76.01–84.00), Q3 (84.01–93.49), and Q4 (≥ 93.50) for women. Models with VAT were best fitted (lower AIC values), with the exception of Metabolic Syndrome for men (Supplementary Table S3). The number of events per variable in the multivariable logistic regression were greater than 10^47^. Multicollinearity between covariates were tested. Weighted regression was used to correct for possible heteroscedasticity. A two-tailed P-value < 0.05 was considered statistically significant. Analyses were performed using SAS version 9.4 (SAS Institute, Inc, Cary, NC).

## Supplementary Information

Supplementary Information

## Data Availability

The data generated during and/or analysed during the current study are available from the corresponding author on reasonable request.
