# Author Correction: Estimated visceral adiposity is associated with risk of cardiometabolic conditions in a population based study

**DOI:** 10.1038/s41598-021-96667-z

**Published:** 2021-08-25

**Authors:** Maria Ruiz‑Castell, Hanen Samouda, Valery Bocquet, Guy Fagherazzi, Saverio Stranges, Laetitia Huiart

**Affiliations:** 1 Department of Population Health, Luxembourg Institute of Health, 1A‑B, rue Thomas Edison, 1445 Strassen, Luxembourg; 2 Nutrition and Health Research Group, Department of Population Health, Luxembourg Institute of Health, 1A‑B, rue Thomas Edison, 1445 Strassen, Luxembourg; 3 Competence Centre for Methodology and Statistics, Department of Population Health, Luxembourg Institute of Health, 1A‑B, rue Thomas Edison, 1445 Strassen, Luxembourg; 4 Deep Digital Phenotyping Research Unit, Department of Population Health, Luxembourg Institute of Health, 1A‑B, rue Thomas Edison, 1445 Strassen, Luxembourg; 5 Department of Epidemiology and Biostatistics, Schulich School of Medicine and Dentistry, Western University, Kresge Building, 1151 Richmond St, London, ON N6A 3K7 Canada; 6 Department of Family Medicine, Schulich School of Medicine and Dentistry, Western University, Western Centre for Public Health and Family Medicine, 1465 Richmond St, London, ON N6G 2M1 Canada

Correction to: *Scientific Reports* 10.1038/s41598-021-88587-9, published online 27 April 2021

The original version of this Article contained errors in the Abstract and the Results section, under the subheading ‘VAT association with all cardiometabolic conditions’, where

“The total adjusted odds ratio (AOR, [95% CI]) for hypertension, prediabetes/diabetes, hypercholesterolemia, and hypertriglyceridemia for the fourth quartile of VAT compared to the lowest were (10.67 [6.95, 16.39]), (6.14 [4.14, 9.10]), (6.03 [3.97, 9.16]) and (9.18 [5.97, 14.12]).”

now reads:

“The total adjusted odds ratio (AOR, [95% CI]) for hypertension, prediabetes/diabetes, hypercholesterolemia, and hypertriglyceridemia for the fourth quartile of VAT compared to the lowest were (10.22 [6.75, 15.47]), (5.90 [4.02, 8.67]), (3.60 [2.47, 5.25]) and (7.67 [5.04, 11.67]).”

“The association observed was strongest in men for hypertension: adjusted OR [95% CI] for men were 2.41 [1.40, 4.14], 3.79 [2.22, 6.47] and 10.80 [6.17, 18.92] for the second, third and fourth quartile of VAT. For women, the values were 1.93 [0.92, 4.02], 3.35 [1.66, 6.74] and 8.20 [4.09, 16.40] for the second, third and fourth quartile of VAT. Nevertheless, women observed a strongest association for combined prediabetes and diabetes (7.11 [3.68, 13.72] for the fourth quartile of VAT in women compared to 5.37 [3.23, 8.93] in men), hypercholesterolemia (8.22 [4.15, 16.26] for the fourth quartile of VAT in women compared to 4.67 [2.72, 8.02] in men) and hypertriglyceridemia (14.97 [6.47, 34.60] for the fourth quartile of VAT in women compared to 7.28 [4.27, 13.40] in men.”

now reads:

“The association observed was strongest in men for hypertension: adjusted OR [95% CI] for men were 2.51 [1.46, 4.29], 4.08 [2.40, 6.93], and 11.83 [6.82, 20.49] for the second, third and fourth quartile of VAT. For women, the values were 1.93 [0.92, 4.00], 3.41 [1.69, 6.85], and 8.21 [4.12, 16.36] for the second, third and fourth quartile of VAT. Nevertheless, women observed a strongest association for combined prediabetes and diabetes (7.57 [3.93, 14.59] for the fourth quartile of VAT in women compared to 5.41 [3.26, 8.97] in men), hypercholesterolemia (5.28 [3.09, 9.00] for the fourth quartile of VAT in women compared to 2.26 [1.33, 3.84] in men) and hypertriglyceridemia (14.62 [6.30, 33.90] for the fourth quartile of VAT in women compared to 6.78 [3.97, 11.56] in men.”

In addition, the original version of this Article contained a repeated error in Table 1, under the headings ‘Lifestyles and socioeconomic characteristics, n (%)’, where

“Aerobic PA, min (median (P25, P75))”

now reads:

“Aerobic PA, min/week (median (P25, P75))”

Finally, in the Methods section, under the subheading ‘Covariates’,

“Lifestyle characteristics included smoking status (current smoking or quit < 12 months vs non-smokers or quit > 12 months), alcohol consumption (non-alcohol consumption, ≤ 6 drinks/week, > 6 drinks/week) and physical activity (aerobic physical activity ≥ 150 min vs aerobic physical activity < 150 min).”

now reads:

“Lifestyle characteristics included smoking status (current smoking or quit < 12 months vs non-smokers or quit > 12 months), alcohol consumption (non-alcohol consumption, ≤ 6 drinks/week, > 6 drinks/week) and aerobic physical activity (min/week).”

The original Article has been corrected.

