# Supplementary material for: Estimated visceral adiposity is associated with risk of cardiometabolic conditions in a population based study
Source: Sci Rep. 2021 Apr 27;11:9121. doi: 10.1038/s41598-021-88587-9 (PMC8079669; doi:10.1038/s41598-021-88587-9)

**Visceral Adiposity and Cardiometabolic Health: Findings from the 2013-2015 EHES-LUX study**

Maria Ruiz-Castell, Hanen Samouda, Valery Bocquet, Guy Fagherazzi, Saverio Stranges and Laetitia Huiart

**Supplementary Table S1. Participants’ Characteristics Stratified by Sex: European Health Examination Survey in Luxembourg, 2013-2015 (N=1 441)**

|  | Total (N=1441) | Men (N=692) | Women (N=749) | P-value^∞^ |
| --- | --- | --- | --- | --- |
|  |  | | |  |
| *Cardiometabolic conditions, n (%)* |  |  |  |  |
| Hypertension | 452 (31.4) | 277 (40.1) | 175 (23.4) | <0.0001 |
| Prediabetes and diabetes | 454 (31.6) | 291 (42.1) | 163 (21.8) | <0.0001 |
| Hypercholesterolemia^†^ | 1002 (69.7) | 506 (73.2) | 496 (66.4) | <0.01 |
| High LDL-Cholesterol^‡^ | 909 (63.1) | 472 (68.2) | 437 (58.3) | <0.01 |
| Low HDL-Cholesterol^∆^ | 433 (30.1) | 226 (32.7) | 207 (27.6) | <0.05 |
| Hypertriglycemia | 421 (29.2) | 278 (40.2) | 143 (19.1) | <0.0001 |
| Metabolic Syndrome | 459 (32.0) | 270 (39.1) | 189 (25.4) | <0.0001 |
| *Cardiometabolic risk factors, median (P25, P75)* |  |  |  |  |
| Systolic blood pressure, mm Hg | 123.5 (112.5, 133.5) | 128.5 (121.5, 137.5) | 116.0 (107.5, 127.5) | <0.0001 |
| Diastolic blood pressure, mm Hg | 79.5 (73.0, 86.0) | 82.5 (75.5, 89.0) | 76.5 (70.5, 84.0) | <0.0001 |
| Total Cholesterol, mg/d | 201.0 (176.0, 226.0) | 202.0 (178.0, 229.0) | 199.0 (175.0, 223.0) | NS |
| HDL-Cholesterol, mg/d | 51.0 (41.0, 60.0) | 44.0 (38.0, 53.0) | 57.0 (48.0, 65.0) | <0.0001 |
| LDL-Cholesterol, mg/d | 126.0 (104.0, 149.0) | 130.0 (107.0, 153.5) | 123.0 (102.0, 143.0) | <0.0001 |
| Triglycerides, mg/d | 93.0 (68.0, 132.0) | 113.0 (80.5, 162.5) | 78.0 (60.0, 108.0) | <0.0001 |
| Fasting plasma glucose, mg/dl | 95.0 (89.0, 101.0) | 98.0 (92.0, 105.0) | 92.0 (87.0, 98.0) | <0.0001 |
| Haemoglobin A1c, mol/mol | 37.0 (34.0, 39.0) | 37.0 (34.0, 40.0) | 37.0 (34.0, 39.0) | NS |
| *Anthropometric Characteristics (median (P25, P75))* |  |  |  |  |
| BMI, kg/m2 | 25.8 (23.1, 29.1) | 26.9 (24.4, 29.4) | 24.8 (21.9, 28.3) | <0.0001 |
| Waist Circumference, cm | 90.0 (81.0, 99.8) | 95.5 (88.5, 104.0) | 84.0 (76.0, 93.5) | <0.0001 |
| Thigh Circumference, cm | 60.0 (56.0, 64.0) | 59.5 (56.0, 63.0) | 60.3 (56.0, 64.8) | <0.05 |
| Predicted visceral adipose tissue (VAT) | 119.9 (74.4, 163.5) | 151.6 (113.8, 196.7) | 89.4 (58.8, 127.4) | <0.0001 |
| *Demographic characteristics (median (P25, P75))* |  |  |  |  |
| Age, years | 45.1 (37.0, 52.9) | 45.3 (37.6, 52.8) | 44.8 (36.4, 53.2) | NS |
| *Lifestyles and socioeconomic characteristics, n (%)* |  |  |  |  |
| Smoking |  |  |  | <0.05 |
| Current smoking or quit < 12 months | 344 (23.9) | 184 (26.6) | 160 (21.4) |  |
| Non-smokers or quit >12 months | 1094 (76.1) | 507 (73.4) | 587 (78.6) |  |
| Alcohol consumption |  |  |  | <0.0001 |
| Non-alcohol (weekly) consumption | 575 (40.0) | 175 (25.3) | 400 (53.6) |  |
| ≤ 6 drinks/week | 455 (31.6) | 221 (31.9) | 234 (31.4) |  |
| > 6 drinks/week | 408 (28.4) | 296 (42.8) | 112 (15.0) |  |
| Aerobic PA |  |  |  |  |
| Aerobic PA>=150 min | 538 (37.4) | 271 (39.2) | 267 (35.7) | NS |
| Aerobic PA, min (median (P25, P75)) | 90.0 (0.0, 240.0) | 90.0 (0.0, 240.0) | 90.0 (0.0, 200.0) | NS |
| Education |  |  |  | NS |
| Primary education | 360 (25.1) | 171 (24.8) | 189 (25.3) |  |
| Secondary education | 549 (38.2) | 255 (37.0) | 294 (39.4) |  |
| Tertiary education | 527 (36.7) | 264 (38.3) | 263 (35.3) |  |
| Working status |  |  |  | <0.0001 |
| Not working | 339 (23.5) | 126 (18.2) | 213 (28.4) |  |
| Working | 1,101 (76.5) | 565 (81.8) | 536 (71.6) |  |

n: Number; Missing values range from 1 to 14 observations; ^†^Hypercholesterolemia was defined as total cholesterol ≥190 mg/dL or on medication to reduce cholesterol. ^‡^High LDL-Cholesterol was defined as blood LDL-C ≥115 mg/dL; ^∆^Low HDL- Cholesterol was defined as blood as HDL-C less than 50 mg/dL for women and 40 mg/dL for men; ^∞^ Pearson's chi-squared test (for probabilities related to frequencies) or Wilcoxon–Mann–Whitney U two-sample test (for probabilities related to medians); NS: Non significant; PA: Physical activity

**Supplementary Table S2. Prevalence of Cardiometabolic Conditions by Waist Circumference Quartiles and Stratified by Sex: European Health Examination Survey in Luxembourg, 2013-2015 (N=1 441)**

|  | Q1 | Q2 | Q3 | Q4 | *P* for trend ^∞^ |
| --- | --- | --- | --- | --- | --- |
| Men, n (%) | 174 | 172 | 176 | 170 |  |
| Hypertension | 35 (20.1) | 53 (31.0) | 70 (39.8) | 119 (70.0) | <0.001 |
| Prediabetes and diabetes | 45 (25.9) | 67 (39.2) | 77 (43.8) | 102 (60.0) | <0.001 |
| Hypercholesterolemia^†^ | 108 (62.1) | 135 (79.0) | 130 (73.9) | 133 (78.2) | 0.004 |
| High LDL cholesterol ^‡^ | 112 (64.4) | 126 (73.3) | 127 (72.2) | 107 (62.9) | 0.75 |
| Low HDL cholesterol ^∆^ | 34 (19.5) | 49 (28.5) | 71 (40.3) | 72 (42.3) | <0.001 |
| Hypertriglycemia | 35 (20.1) | 60 (34.9) | 73 (41.5) | 110 (64.7) | <0.001 |
| Metabolic Syndrome | 0 (0.0) | 28 (16.5) | 105 (59.7) | 137 (80.6) | <0.001 |
|  |  |  |  |  |  |
| Women, n (%) | 196 | 189 | 177 | 187 |  |
| Hypertension | 22 (11.3) | 35 (18.6) | 41 (23.3) | 77 (41.4) | <0.001 |
| Prediabetes and diabetes | 20 (10.2) | 30 (16.0) | 42 (23.9) | 71 (38.0) | <0.001 |
| Hypercholesterolemia^†^ | 110 (56.1) | 117 (62.2) | 128 (72.7) | 141 (75.4) | <0.001 |
| High LDL cholesterol ^‡^ | 96 (49.0) | 104 (55.0) | 112 (63.3) | 125 (66.8) | <0.001 |
| Low HDL cholesterol ^∆^ | 27 (13.8) | 37 (19.6) | 58 (32.8) | 85 (45.4) | <0.001 |
| Hypertriglycemia | 6 (3.1) | 26 (13.8) | 42 (23.7) | 69 (36.9) | <0.001 |
| Metabolic Syndrome | 0 (0.0) | 19 (10.1) | 64 (36.6) | 106 (57.3) | <0.001 |

n: Number; Missing values range from 1 to 14 observations; ^†^Hypercholesterolemia was defined as total cholesterol ≥190 mg/dL or on medication to reduce cholesterol. ^‡^High LDL-Cholesterol was defined as blood LDL-C ≥115 mg/dL; ^∆^ Low HDL cholesterol was defined as blood as HDL less than 50 mg/dL for women and 40 mg/dL; ^∞^Probability estimated by Cochran-Armitage trend test. Q1 (≤ 88.50), Q2 (88.51-95.45), Q3 (95.46-103.99), and Q4 (≥104.00) for men and Q1(≤76.00), Q2 (76.01-84.00), Q3 (84.01-93.49), and Q4 (≥ 93.50) for women.

**Supplementary Table S3. Akaike Information Criterion Values of Anthropometrically Predicted Visceral Adiposity and Waist Circumference Univariate Models: European Health Examination Survey in Luxembourg, 2013-2015 (N=1 441)**

| Cardiometabolic condition | AIC values for VAT | AIC values for WC |
| --- | --- | --- |
| Men |  |  |
| Hypertension | 869.7 | 889.6 |
| Prediabetes and diabetes | 932.5 | 959.1 |
| Hypercholesterolemia | 849.4 | 864.1 |
| Hypertriglycemia | 905.2 | 918.8 |
| Metabolic Syndrome | 651.3 | 599.3 |
| Women |  |  |
| Hypertension | 660.6 | 707.2 |
| Prediabetes and diabetes | 653.9 | 692.0 |
| Hypercholesterolemia | 828.5 | 883.1 |
| Hypertriglycemia | 564.9 | 602.8 |
| Metabolic Syndrome | 545.6 | 566.6 |

VAT : Anthropometrically-predicted visceral adipose tissue; WC : Waist Circumference; AIC: Akaike information criterion (lower AIC value gives the best fit model)

**Supplementary Figure S1**: Relationship between Estimated VAT and Thigh Circumference with Waist circumference and age constant.


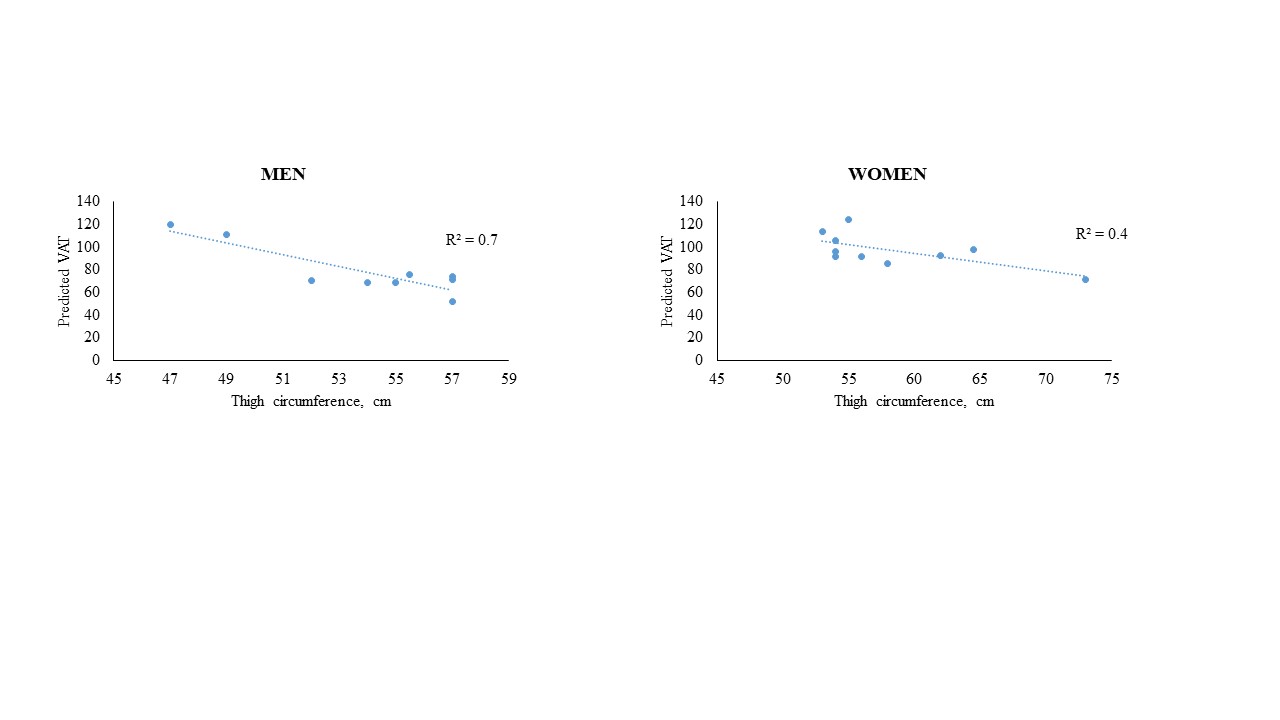

Supplement: Supplementary file 1 — Supplementary Information [file 41598_2021_88587_MOESM1_ESM.docx]
